# Patient experiences with hereditary cancer genetic test result release in the setting of the 21st Century Cures Act final rule

**DOI:** 10.1007/s10689-026-00613-9

**Published:** 2026-10-27

**Authors:** Cameron Bell, Samantha Pipito, Ashika Mani, Arravinth Anantharajah, Taylor Apostolico, Angela G. Arnold, Jacqueline Cappadocia, Dana Farengo Clark, Melissa Heller, Jessica M. Long, Derek Mann, Emma McKennon, Joanna Mercado, Jacquelyn Powers, Anna Raper, Kelsey Spielman, Bryson W. Katona, Kara Maxwell, Katherine L. Nathanson, Mersedeh Rohanizadegan, Payal D. Shah, Angela Bradbury, Susan M. Domchek, Rebecca Mueller, Jessica E. Ebrahimzadeh

**Affiliations:** 1 Master of Science in Genetic Counseling Program, Perelman School of Medicine, University of Pennsylvania, PA Philadelphia, USA; 2 Division of Hematology/Oncology, Department of Medicine, University of Pennsylvania Health System, Philadelphia, PA 19104 USA; 3 Department of Biostatistics, Epidemiology and Informatics, Perelman School of Medicine, University of Pennsylvania, Philadelphia, PA USA; 4 Division of Translational Medicine & Human Genetics, Department of Medicine, University of Pennsylvania Perelman School of Medicine, Philadelphia, PA USA; 5 Division of Gastroenterology and Hepatology, Department of Medicine, University of Pennsylvania Perelman School of Medicine, Philadelphia, PA USA

**Keywords:** 21st Century Cures Act, Hereditary cancer genetic testing, Genetic counseling, Electronic health record (EHR), Results communication, Patient-centered care

## Abstract

The 21st Century Cures Act Final Rule is a federal law recently implemented in the United States of America which prompted a practice shift among genetic counselors who historically disclosed genetic testing results by directly speaking with patients. Herein, we report participant experiences following a single-center’s approach of patients selecting provider-facilitated versus immediate release of hereditary cancer genetic testing results in the electronic health record preceding provider review. A voluntary survey was sent to patients who completed pre-test genetic counseling and hereditary cancer genetic testing within the University of Pennsylvania Health System. Responses were analyzed using descriptive statistics. Participants (*n* = 153) were predominately White (77%), female (81%), obtained Master’s or Doctoral degrees (47%), and a median age of 60 (range 22–81). Participants across both result release cohorts did not differ in demographics, indication for testing, answers to knowledge-based questions or initial emotional responses. When asked about release preference for future genetic testing, most participants preferred provider-facilitated electronic portal message or a phone call. Of the participants who initially elected immediate release, only 18% (20/113) would again elect immediate release for future genetic tests. Participants across results release cohorts predominately elect for provider-facilitated release for future genetic testing, suggesting provider involvement may benefit patient disclosure experience.

**Supplementary Information:**

The online version contains supplementary material available at https://doi.org/10.1007/s10689-026-00613-9.

## Introduction

The 21st Century Cures Act is a federal law in the United States of America which was signed into law on December 13, 2016 and entered the compliance phase on April 5, 2021. The primary goal of the law is to improve interoperability of health records in support of medical product development and to bring advances to patients. This law also aims to make electronic health records (EHRs) rapidly and fully accessible to patients. The Information Blocking Rule of the Cures Act mandates that patients have the ability access their health information when they want, without waiting for their healthcare provider (HCP) to communicate results to them. Following its enactment, immediate release of results has been reported as a nearly universal preference among patients. In one study of over 8000 patients who had received various clinical test results (including but not limited to unspecified blood tests, imaging/biopsies, and COVID-19 testing) through their online patient portal accounts, 96% of respondents indicated that they preferred this method of result over having a HCP disclose them [1]. Notably, respondents with abnormal results more frequently reported feeling worry (16.5%) compared to respondents with normal results (5%) [1]. The preference for immediate release has been demonstrated repeatedly in patients receiving their results without HCP intervention, along with lower levels of comprehension and subsequently increased requests for further explanations, generating concern over potentially increased demand on providers [2–7]. While the overall positive reception among patients has been well documented, multiple recent studies of patients and healthcare providers have indicated particular concern over the release of highly sensitive or distressing information, such as biopsy or radiology results [8–11]. Similarly, a 2022 survey of over 8000 patients at a large regional medical center detailed variable patient preference for/against immediate result release based on the test type and perceived sensitivity of the results; while 57% of respondents indicated that result release via online portal is acceptable for a strep test, only 17% of respondents felt that this was acceptable for cancer testing [12].

Importantly, there have not been any published studies about patient experiences with release of genetic test results through the EHR. While genetic testing has similarities to other medical tests, there are characteristics that add to the complexity and sensitivity of learning results. Germline genetics are immutable, and test results may be complex, predictive, identifying, and potentially stigmatizing [13, 14]. In a traditional genetic counseling model, genetic test results were disclosed by directly speaking to patients at a follow up visit in person, which eventually evolved to telephone and telehealth disclosures [15, 16]. Therefore, immediate release of genetic results through the EHR prompted yet another practice shift for genetic counselors.

Adaptation of the 21st Cures Act Final Rule vary across health systems among the genetics community due to patient portal software, EHR capabilities, as well as interpretation of the law. Many genetic counselors and physicians order testing through third-party laboratories and manually scan genetic test results into the EHR, allowing HCP review prior to releasing results to patients through a portal. Other organizations have internal genetic testing labs or third-party laboratories integrated with the EHR, allowing the genetic test results to be immediately released to the patient portal upon completion. Implementation for results release across health systems also differ, with many accommodating the law by placing all new results and notes immediately into the patient portal, whether the patient has asked for them or not. This is not what the law requires, but rather a choice made by health systems since it’s a simple way to achieve compliance. Further, there are varying interpretations about the definition of “reasonable delay” or the allowable exceptions to delay, creating differences in the manner results are released across health systems [17]. Importantly, the law allows case-by-case implementation for results release, including patient preference.

This study examines a single center’s approach of including result disclosure options as a part of pre-test hereditary cancer genetic counseling. The center’s integration of genetic test results into the EHR enabled options including: (1) immediate release of test results through the EHR preceding provider review or (2) provider-facilitated result disclosure prior to results release in the portal. Post-test counseling was always offered regardless how participants first received results. Herein, we report participant demographics and indications for testing based on release preferences. We also report on experiences, emotional responses, and answers to knowledge-based questions based on how participants first received their results.

## Materials and methods

### Genomic integration and clinical workflow

Penn Medicine Center for Genomic Medicine (PMGM) at the University of Pennsylvania Health System collaborated with EPIC to innovate the integration of genomic medicine into the EHR. One specific element of this initiative included automatically uploaded results in the EHR with discrete genetic data that were linked from test orders placed in the EHR [18]. Therefore, the genetic test results from integrated orders appear immediately in patient portals, similar to other routine lab tests like a complete blood count.

As part of routine clinical care, genetic counselors provided the option for provider-facilitated release or immediate release at the time of pre-test hereditary cancer genetic counseling. Individuals that elect for immediate release still received asynchronous post-test counseling. In addition to result release, genetic counselors also reviewed options for post-test counseling, such as phone versus appointment. Of note, attempts to contact were often within a couple of business days of results receipt. Discussions around result release preferences and post-test counseling were not scripted and varied across genetic counselors. Participant preferences were documented in the templated visit note. At the time of placing the EHR integrated genetic test order, the genetic counselor was required to select “immediate release” versus “manual-release only”, along with the reason for manual release (Online Resource 1). The default selection of “immediate” was removed to ensure that the release method would accurately reflect the participant’s election. Participants were also sent an after-visit summary through the patient portal, outlining the disclosure plan.

Completed genetic test results appear in both the “Labs” and customized “Precision Medicine” tabs, with positive results linked to a genomic indicator to allow for more transparent access to genetic information that may guide medical management. Those that selected “immediate release” had the lab report instantly available in the patient portal at the same time providers could access the results in the EHR; no additional patient directed language or interpretation was provided at the time of immediate release. Those that selected “manual-release” had results accessible in the EHR to all providers, but were only viewable in the patient portal after a provider manually selected a button to release the results (Online Resource 1). Genetic counselors attempted to review results with all participants primarily by phone, regardless of initial release preference or result type. If patients were unable to be reached, a summary letter reviewing results and interpretation were sent via patient portal (if active) or postal mail.

### Survey

A voluntary survey was sent to individuals undergoing hereditary cancer genetic testing at the University of Pennsylvania Health System for whom results were released through EHR integrated labs between September 2024–February 2025. To be eligible for the survey, participants needed to be able read English, be 18 years or older at the time of recruitment, and be enrolled in the electronic patient portal. Individuals were invited to participate in the survey via direct electronic patient portal message sent by study personnel within 2 weeks of their genetic test result release. Patients were consented electronically through REDCap. There were no reminders and no compensation for completing the survey. For the purposes of this study, survey participants who indicated that they had genetic testing for indications other than hereditary cancer or those without pre-test genetic counseling were further excluded from our study cohort.

The survey was developed by the investigative team and included questions that were adapted from a previously published study and a widely used questionnaire about genetic test results [1, 19]. It consisted of 28 questions including nine experiential, six demographic, five psychosocial, and four knowledge-based questions (Online Resource 2). There were additional branch logic questions for those who self-reported a variant of uncertain significance or received results via immediate release. The survey included questions that were adapted from an instrument previously used to assess patient perspectives on immediate test result release through an electronic patient portal that was adapted from a validated measure [1]. Questions regarding patient feelings about genetic test results were adapted from the validated Feelings About genomiC Testing Results (FACToR) Questionnaire [19]. To interpret participant responses to seven questions assessing participant comprehension of the utility, meaning, and limitations of their test results, we validated by reviewing participant’s genetic test reports. (Online Resource 2, questions #4–10). Actual result type and concordance/discordance of participant response were entered and scored in REDCap. Genetic counseling notes were reviewed when responses were discordant to provide additional clarification. Additionally, participant responses to the knowledge-based questions within the survey were designated as “correct” or “incorrect” with REDCap based on their recorded result type; negative, VUS and positive results had a different answer key to reflect the “correct” response. To score participant responses to a question regarding how they would like to first learn genetic test results in the event they pursued additional genetic testing in the future, we grouped results as either provider-facilitated release or immediate release. Provider-facilitated release was defined as those who selected to first receive their results on patient portal with a message from their provider, phone call from their provider, or appointment with their provider. Immediate release was defined as those who selected the preference to view results on patient portal without any provider review or explanation.

Differences in continuous variables for the cohorts were analyzed utilizing two-sample t-tests, and categorical variables were analyzed utilizing chi-square tests of independence. All tests were two-sided, with a significance level set at α < 0.05. We performed inductive content analysis on responses to a single free-response question (#25, Online Resource 2) which asked participants, “Is there anything else you’d like to share regarding your experience with receiving your genetic test results?”

Participants were ascertained from five Hospital of the University of Pennsylvania Health System sites. The institutional review board (IRB) at the University of Pennsylvania approved all study procedures, as did the Princeton Health Scientific Review Committee (PHSRC), the Chester County Hospital Scientific Review Committee (CCHSRC), and the University of Pennsylvania’s Clinical Trials Scientific Review and Monitoring Committee (CTSRMC). These reviewing bodies additionally granted waivers of written documentation of informed consent for survey participants.

## Results

### Survey population characteristics

**Fig. 1:**
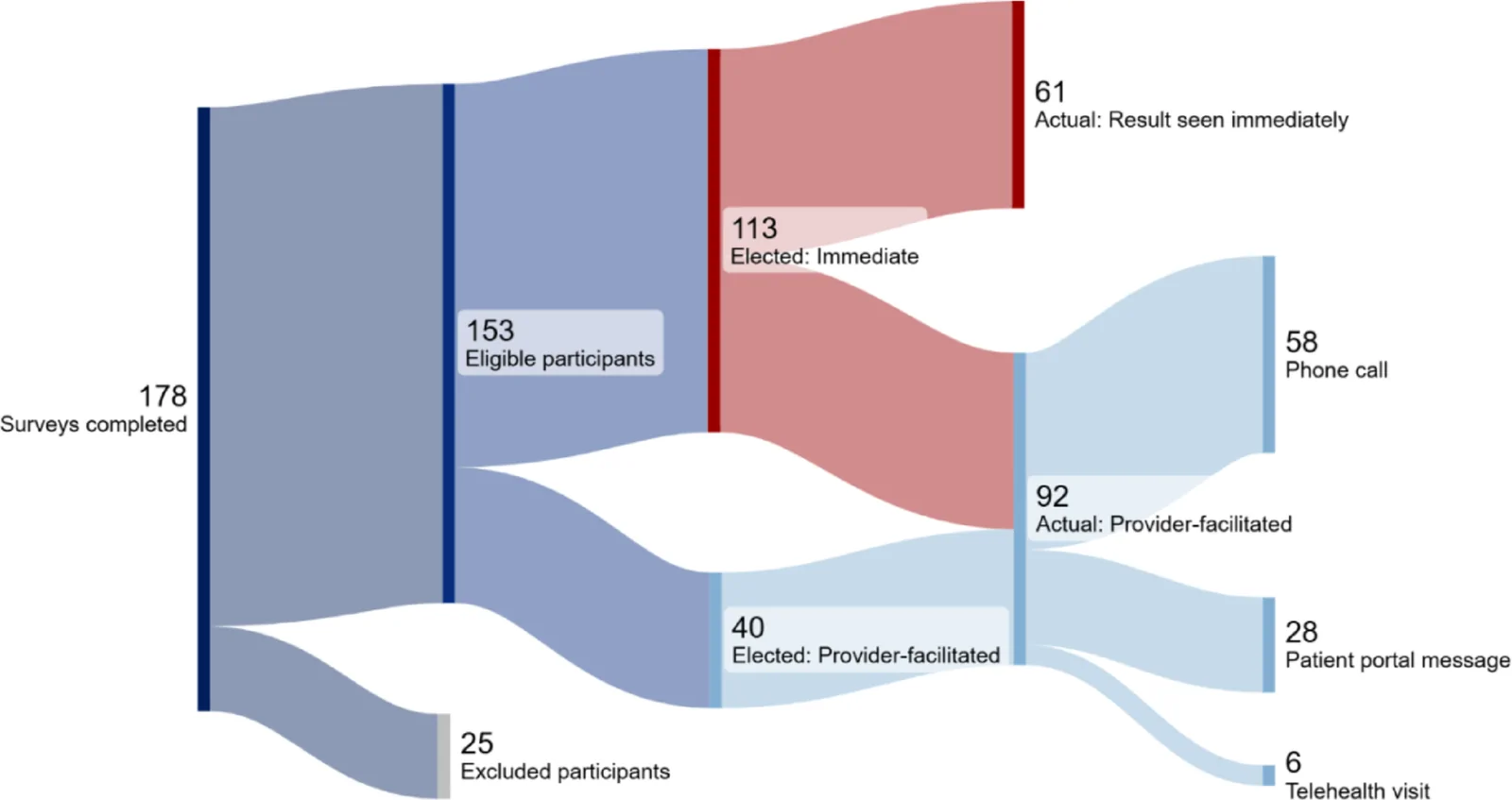
This diagram depicts the breakdown of survey participants based on both preferred and actual result release methods. This also includes how results were first learned when provider-facilitated, including phone call, patient portal message or telehealth visit. The elected release preference was determined via clinical documentation from chart review. The actual result release type was determined via survey response. The reason for the difference between elected and actual result release was due to our clinical workflows; genetic counselors attempt to review results with all patients, regardless of initial release preference or result type. Created using SankeyMATIC

Over 1500 surveys were sent to individuals who had undergone hereditary cancer genetic testing; 178 of 1587 completed the survey, resulting in a response rate of 11.2%. Twenty-five survey participants were excluded due to completing genetic testing for indications other than hereditary cancer or because they did not complete pre-test genetic counseling, leaving 153 responses analyzed (Fig. 1). Most participants identified as female, White, holding a at least a four-year college degree, and earning at least $50,000 per year (Table 1). 113 participants (74%) elected for immediate release and 40 participants (26%) elected for provider-facilitated release. Despite immediate release preference, some participants received results from a genetic counselor prior to seeing their results in the portal due to clinical workflow. Therefore, 92 participants (60%) first learned about their results from a provider, whereas 61 participants (40%) first saw their results in patient portal without provider involvement (Fig. 1). Of those with provider-facilitated release, 63% (58/92) were reviewed over the phone, 30% (28/92) received a patient portal message, and 7% (6/92) had a telehealth visit. 108 participant results (71%) were negative, 28 (18%) had likely pathogenic or pathogenic variants (LPV/PV), and 17 (11%) had a variant of uncertain significance (VUS).

There were no significant differences in participant self-reported demographics (race, gender, age, formal education, work experience in healthcare and household income) between the provider- facilitated preferred and immediate release preferred cohorts (Table 1). There were also no significant differences in indication for testing between the provider-facilitated preferred and immediate release preferred cohorts.

**Table 1:**
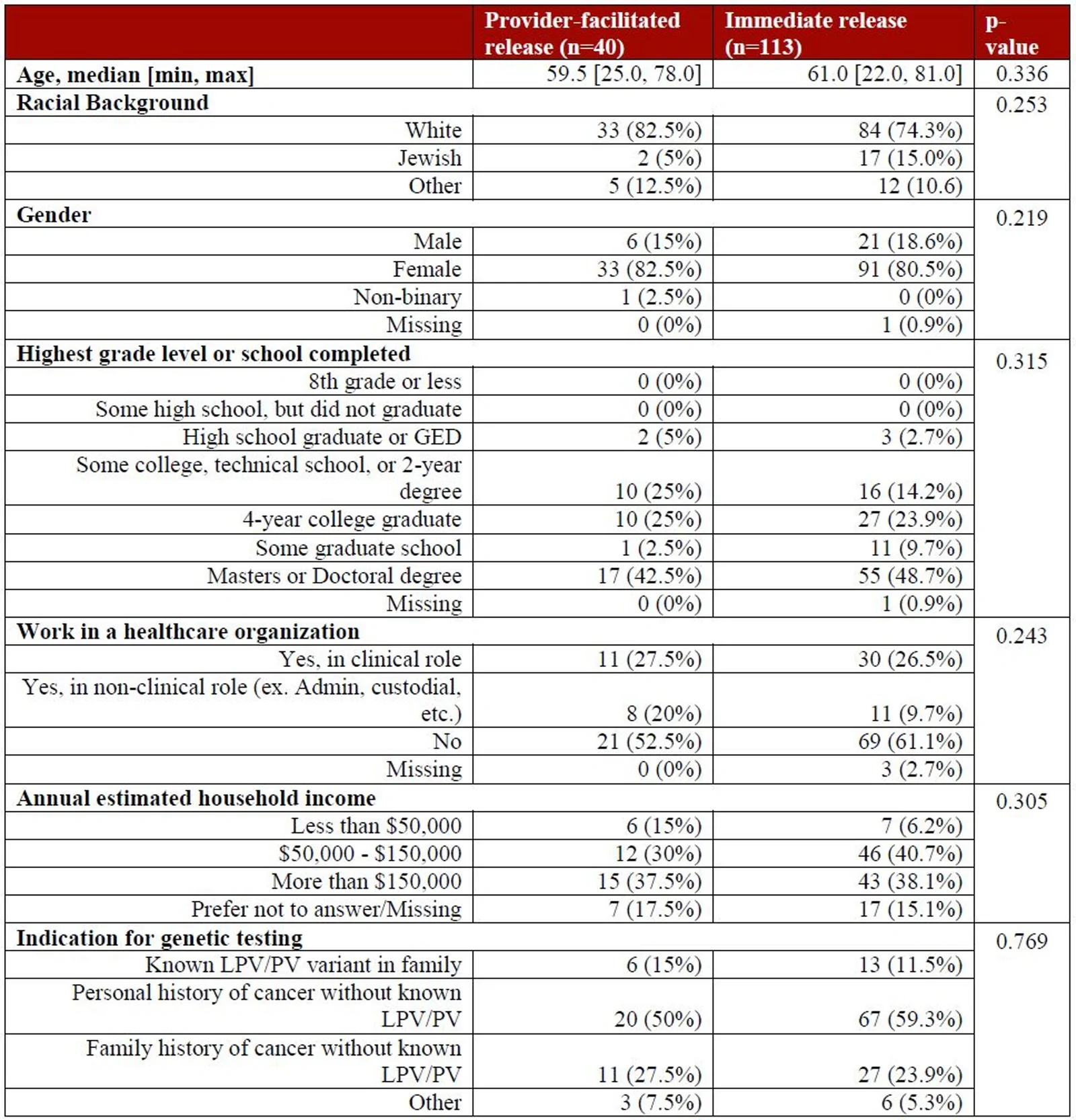
Participant characteristics

No significant differences were found among participant demographic and clinical characteristics when comparing between release preferences (provider-facilitated release vs. immediate release)

### Knowledge-based responses

**Fig. 2:**
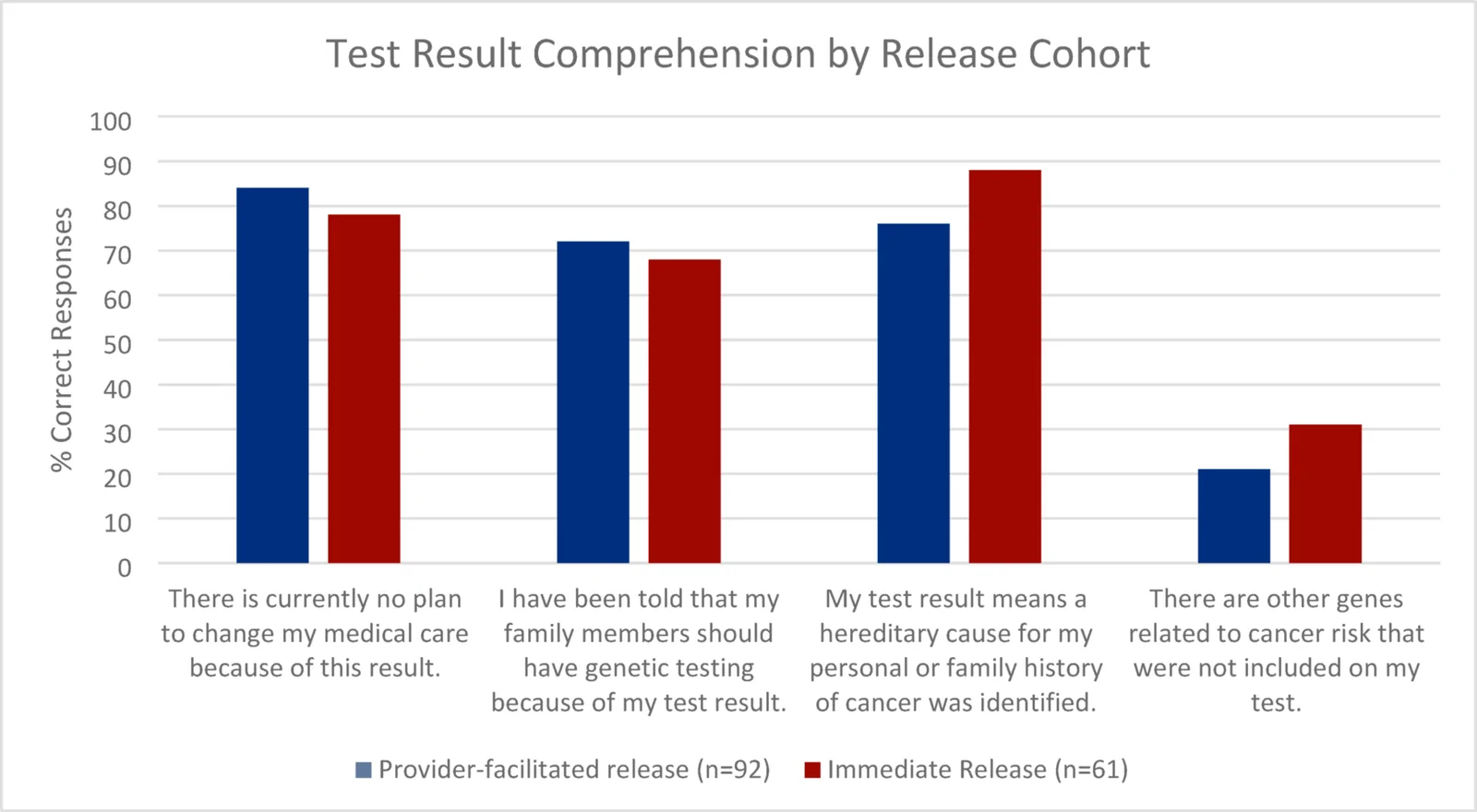
Distribution of correct/incorrect answers to genetic test result comprehension questions by result release cohort; the answer key differed based on result type to determine the correct response. No significant differences were found in participants’ ability to answer these questions correctly when compared between result release cohort (provider-facilitated vs. immediate release). Created using Microsoft Excel

When stratified by method of result release, no significant relationships were found in participants’ ability to answer knowledge questions correctly (Fig. 2). The rate at which participants correctly self-reported their result was similar between result release cohorts, with an accuracy of 90% among those who had a genetic counselor disclose their results and an accuracy rate of 92% among those who received their results immediately through the patient portal. Of note, 7/17 (41%) of participants with a verified VUS result, self-reported a negative result. Questions #8–10 were included with branching logic and only appeared to participants who self-reported a VUS result, which yielded a total of only 10 responses to these questions. Importantly, none of the participants with an isolated VUS result self-reported a positive result.

### Emotional responses

Answers to the initial emotional response questions were first stratified by self-reported result type. Participants’ reported levels of relief were significantly higher among those with negative results, with 65/112 (58%) indicating they felt “a great deal” of relief while the majority (15/26, 58%) of the positive group answered, “not at all” (*p* = < 0.001). Similarly, when asked about levels of anxiety, 9/26 (35%) of the positive group indicated that they felt “a good/great deal” of anxiety while 80/112 (71%) of the negative group answered, “not at all” or “a little” (*p* = 0.015). The remaining questions regarding uncertainty about the participants’ and their families’ risks of disease and clarity of choices for care did not yield any statistically significant differences between reported results cohorts.

**Fig. 3:**
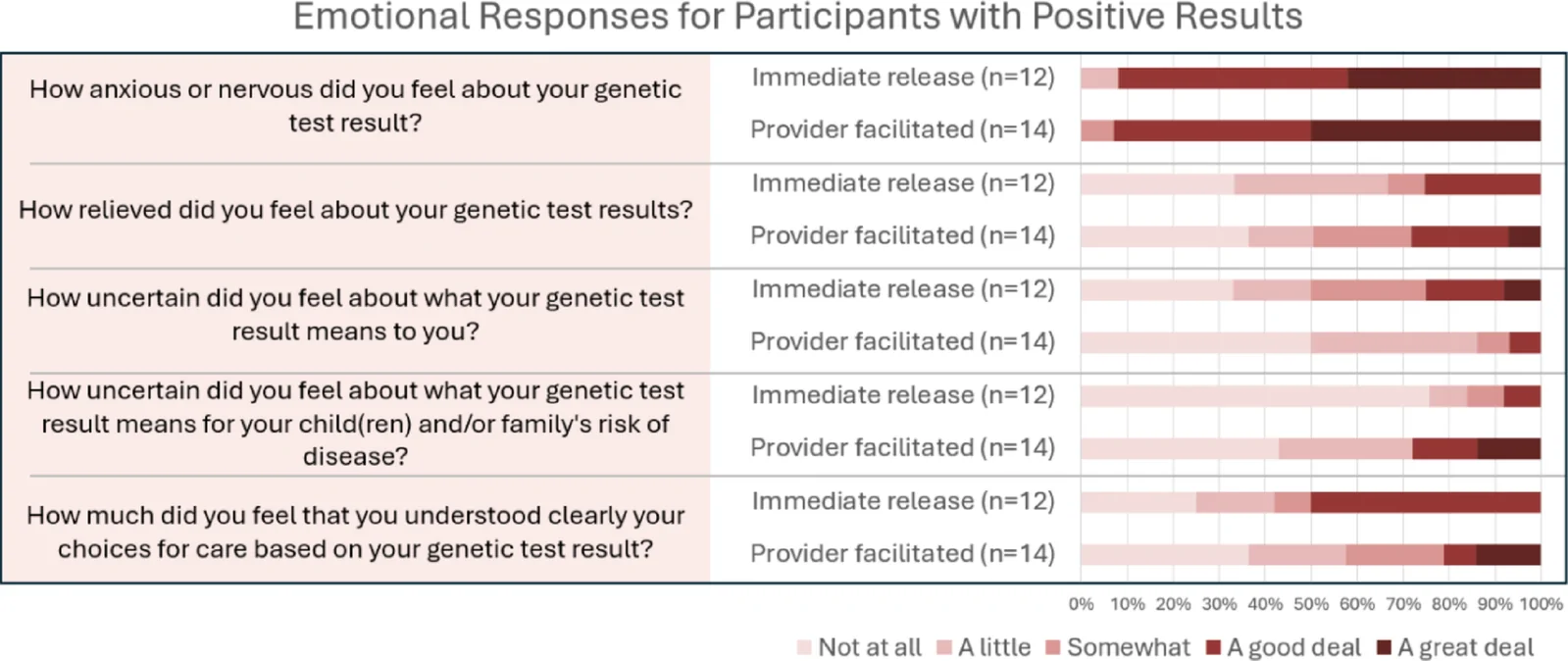
Emotional responses in participants with positive results. Participant responses to questions assessing their initial feelings (anxiety, relief, uncertainty and clarity of future options) pertaining to test results, including only those with positive results and separated by result release cohort. No significant relationships were found in participants’ ratings of emotional response when comparing between release cohort (provider-facilitated release vs. immediate release)

When stratified by result release method (provider-facilitated release vs. immediate release), no significant differences were found among participant emotional response answers. Participants with positive genetic test results with immediate release more frequently responded with “a good/great deal” of anxiety (6/12, 50%) compared with result-positive participants who had a provider-facilitated disclosure (3/14, 21%), though did not reach statistical significance (*p* = 0.128) (Fig. 3).

### Experiential responses

**Fig. 4:**
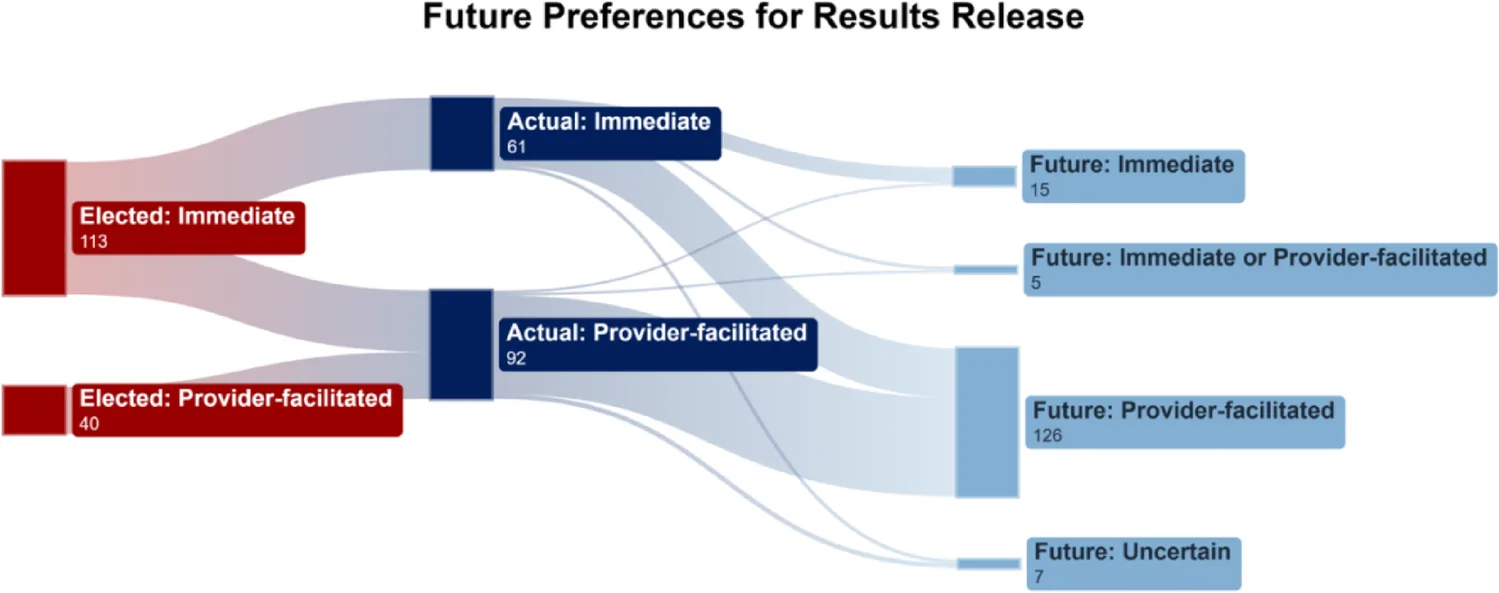
Participants responses when asked how they would like to first learn about their results if they had a reason to pursue additional genetic testing in the future. Figure includes participants selected results release, actual results release and future preferences for results release. Participants could choose more than one option. Created using SankeyDiagram.ai

All participants who opted for provider-facilitated disclosure (40/40, 100%) would maintain provider involvement when first learning their results in the setting of future genetic testing. In contrast, only 18% (20/113) of participants who initially selected immediate release would again elect to receive results first in the portal without provider review. Notably, not all participants received the results based on their preference. Of those who actually received results via immediate release, 21% (13/61) would again opt to learn their results first without provider review, 5% (3/61) responded that they would consider immediate release or provider-facilitated disclosure, 69% (42/61) would elect for provider-facilitated release, and 5% (3/61) were uncertain (Fig. 4).

The most common preference for future genetic test results release was to receive results in the patient portal along with a message from their provider (67/153, 44%) or a phone call from their provider (66/153, 43%). When stratified by result type, there were no significant relationships regarding preferred future disclosure methods.

Participants were provided with the option to share their thoughts on the experience of receiving their genetic test result in a free text field. 65/153 (42%) of participants provided responses to this question, often providing positive feedback about their genetic counselor (23/153) or commenting on clinical logistics or the practical impact of genetic test results (18/153). Twenty-two participants provided deeper reflection on their experience receiving and interpreting their genetic test results which led to three content categories. Content categories are described and illustrated with relevant quotes in Table 2.

**Table 2:** Content categories and illustrative quotes from the open-ended survey question that asked, “Is there anything else you'd like to share regarding your experience with receiving your genetic test results?”

Content category	Illustrative quotes	*N*
Valuing provider-facilitated option	*“Having some kind of face-to-face on telehealth was very helpful. I think just reviewing them on my own without my genetic counselor would’ve been more upsetting and confusing*,* even though she explained to me what a positive test means before I tested.”* *“I was very appreciative of the option to withhold [patient portal] notification until a knowledgeable provider could review them.”*	8
Valuing immediate-release option	*“I really appreciate learning as soon as results were available that they were negative*,* and then having the opportunity to understand them in more detail at a later date in a phone call with the genetic counselor.”* *“I would want to receive the results in the manner they are first available to me [and] not delaying sharing results if a provider wasn’t available to talk with me immediately.”*	7
Experiencing uncertainty from immediate-release	*“My genetic counselor warned me not to google*,* but I still did. My portal result made me think it was positive for [a] mutation*,* but the counselor [said] the mutation was not significant and not to worry. I messaged her and she called within the hour. I would normally talk to my dad about this*,* and he’d talk me down*,* but he’s the one who died from pancreatic cancer*,* so I spiraled.”* *“The test result on [patient portal] read “positive” - which made me panic. However*,* it was [positive] for APC [p.I1307K]- and not for Lynch*,* which was my main concern. I was very grateful to receive a call from my genetic counselor shortly after to help clarify that I was negative for the big scary thing… It would have been very helpful for the test results to have been divided so that I immediately knew I was negative for Lynch.”*	7

## Discussion

The implementation of the Cures Act Information Blocking Rule has prompted a practice shift for genetic counselors facilitating genetic testing. Our institution adapted to this law by providing patients result disclosure options as part of pre-test hereditary cancer genetic counseling. Options included immediate release of results through the electronic patient portal or provider-facilitated disclosure with delayed portal release. Through the surveyed participants, we compared participant demographics and indications for testing based on release preferences. We also compared emotional responses, answers to knowledge-based questions, and experiences based on how participants received their results. While we do not report significant differences in any of these areas based on result release cohort, participants express benefitting from workflows that allow them to opt into or out of immediate result release.

Comparison of answers to knowledge-based questions across both release cohorts in this study demonstrates no significant difference in understanding based on how participants first learned about their genetic test results. Notably, most participants with immediate release (43/61, 70%) had spoken to a genetic counselor post-test prior to taking this survey. Likewise, our comparison of perceived vs. actual result type among both populations showed almost no difference in result comprehension, with accuracy rates of 90% and 92% in the provider-facilitated and immediate cohorts, respectively. This could indicate that timing of provider involvement in result disclosure does not have an impact on the patient’s understanding of the result.

We assessed participants’ reported feelings of anxiety, relief, and uncertainty when they first learned their genetic testing results. Among participants with immediate release, 6/12 (50%) of positive result participants reported feeling “a good/great deal” of anxiety when they first learned about their results compared to only 3/14 (21%) of positive participants in the provider-facilitated cohort, though this difference did not reach statistical significance. Previous studies evaluating whether immediate result release elevates patient anxiety have mixed results among HCPs and patients alike, however it is largely agreed upon that anxiety is more frequently reported among immediate release patients whose results were positive or particularly complex [1, 2, 12, 20]. Our results suggest that the main predictor of patient anxiety related to hereditary cancer genetic testing is a positive genetic test result. The release method did not show any significance in patient anxiety when individuals first learned their results, although this may be due to the small size of the cohort.

Despite few significant differences in knowledge-based questions and initial emotional responses to genetic testing results between release cohorts, our study showed that most participants in both cohorts would elect provider-facilitated disclosure if they were to undergo genetic testing again in the future. Of the immediate release participants who indicated that they would prefer future provider-facilitated disclosure, 45% (51/113) indicated that they would opt for a provider message in the portal accompanying their results at the time of release, followed by 32% (36/113) who stated preference to first learn results over the phone. Notably, only 18% (20/113) of immediate release participants indicated a preference for immediate release again in the future. This change in preference may be related to the options that their genetic counselor provided at the time of pre-test visit. While conversations to determine disclosure preference were not scripted, many genetic counselors provided the option for immediate portal release versus a phone call; the option for participants to receive the results in the portal with a provider message after the provider had a chance to review them was not offered routinely. This may indicate that participants value the HCPs interpretations at the time of results release. Other studies have also demonstrated that patients with immediate release are generally enthusiastic about the convenience of immediate methods, but often want more resources or actionable information about their results [21].

The scope of this study did not formally assess the opinions of this result release approach among genetic counselors. Within our genetic counseling group, we found providing patients with an option for genetic result release was an easy addition to a pre-test counseling session. Documentation was templated into our visit note, in addition to selecting “immediate” versus “manual-release only” within the EHR integrated order (Online Resource 1). Further, allowing this selection is consistent with the patient-centered values of genetic counseling which empowers patients to decide how they prefer to proceed with next steps and results. Participants who opted for provider-facilitated and immediate release both provided feedback in this survey, sharing they appreciated being given options and the opportunity to choose their preferred method of results disclosure.

One limitation of this study was that 52 of 113 participants that opted for immediate release ended up having provider-facilitated release. This was due to our clinical workflow which involved post-test counseling regardless of result release preference. Some participants may not routinely check or get alerts for their patient portal, which yielded in them learning about their results more promptly from their genetic counselor. Another limitation is the conversation regarding results release and post-test counseling preferences were not scripted and varied across genetic counselors; documented participant preferences were a result of shared-decision making, but the decision could have been influenced by the provider. This study was also limited by many participants completing the survey after speaking with a genetic counselor, which may not capture initial reactions for those who elected immediate release. None of the survey questions required answers, and there were some questions which only appeared for certain participants based on branching logic, leading to some inconsistencies in survey completion. Further, the response rate was 11.2%; there was a lack of diversity in the respondents with regard to race, gender, income and education level, which may not accurately represent the individuals our tertiary medical center serves. The potential for bias in this self-selected population must be considered. Furthermore, the low response rate resulted in a small total number of study participants, warranting further investigations with larger, more diverse cohorts. Future studies should investigate result disclosure preferences and experiences in more diverse groups, across other genetic specialties, and among populations that are not receiving pre-test education from a genetic counselor.

Overall, our study found demographic and clinical indications were similar between the release preference cohorts. We also found similarities between results release cohorts and knowledge-based questions and emotional reactions. Importantly, our study suggests many patients prefer learning genetic testing results from a HCP either with a message in the patient portal or through a phone call. HCP involvement may also be more beneficial to patients with positive genetic testing results, who indicated more feelings of anxiety and uncertainty than those with negative or uncertain results. Notably, most participants who opted for immediate release and all participants with provider-facilitated release would elect for provider-facilitated release for future genetic testing, suggesting provider involvement may benefit patient experience. As genetic counseling service delivery models continue to evolve, it is important to further define the impact of immediate portal release to inform policies, especially given that our approach of allowing patients to select preferred approach for disclosure is feasible, efficient, and valued by survey respondents.

## Supplementary Information

Below is the link to the electronic supplementary material.

Supplementary Material 1

## Data Availability

Data are available from the authors upon request.
