# Supplementary material for: Patient experiences with hereditary cancer genetic test result release in the setting of the 21st Century Cures Act final rule
Source: Fam Cancer. 2026 Oct 27;25(4):99. doi: 10.1007/s10689-026-00613-9 (PMC13714062; doi:10.1007/s10689-026-00613-9)
Supplement: Supplementary file 1 [file 10689_2026_613_MOESM1_ESM.pdf]

Supplemental materials for submission to Familial Cancer of the following research article:

Title: Patient Experiences with Hereditary Cancer Genetic Test Result Release in the Setting of the 21st Century Cures Act Final Rule

Authors: Cameron Bell[1], Samantha Pipito[2], Ashika Mani[3], Arravinth Anantharajah[2], Taylor Apostolico[2], Angela G. Arnold[2], Jacqueline Cappadocia[2], Dana Farengo Clark[2], Melissa Heller[2], Jessica M. Long[2], Derek Mann[2], Emma McKennon[2], Joanna Mercado[2], Jacquelyn Powers[2], Anna Raper[4], Kelsey Spielman[2], Bryson W. Katona[5], Kara Maxwell[2], Katherine L. Nathanson[4], Mersedeh Rohanizadegan[4], Payal D. Shah[2], Angela Bradbury[2], Susan M. Domchek[2], Rebecca Mueller[1], Jessica E. Ebrahimzadeh[2]

[1] Master of Science in Genetic Counseling Program, Perelman School of Medicine, University of Pennsylvania;

[2] Division of Hematology/Oncology, Department of Medicine, University of Pennsylvania Health System, Philadelphia, PA, USA;

[3] Department of Biostatistics, Epidemiology and Informatics, Perelman School of Medicine, University of Pennsylvania, Philadelphia, PA, USA;

[4] Division of Translational Medicine & Human Genetics, Department of Medicine, University of Pennsylvania Perelman School of Medicine, Philadelphia, PA, USA;

[5] Division of Gastroenterology and Hepatology, Department of Medicine, University of Pennsylvania Perelman School of Medicine, Philadelphia, PA, USA

Corresponding author: **Jessica E. Ebrahimzadeh**

Corresponding author's contact information:

Division of Hematology/Oncology

Department of Medicine

University of Pennsylvania Health System

Philadelphia, PA 19104

jebra@pennmedicine.upenn.edu

215-349-5473

Online Resource 1:

A. Screenshot of test result release option in EPIC electronic health record. Most tests in EPIC are defaulted to "Immediate" release. At our institution, all germline genetic test results had the "Immediate" release to patient default removed. This field is still required, but ordering clinician has to select between "Immediate" and "Manual release only." For those that are manual release, the clinician has to choose the reason for preventing automatic release. For our purposes, we selected "Patient or legal rep agreed to withhold or delay" given that the patient was provided with a choice of results release.

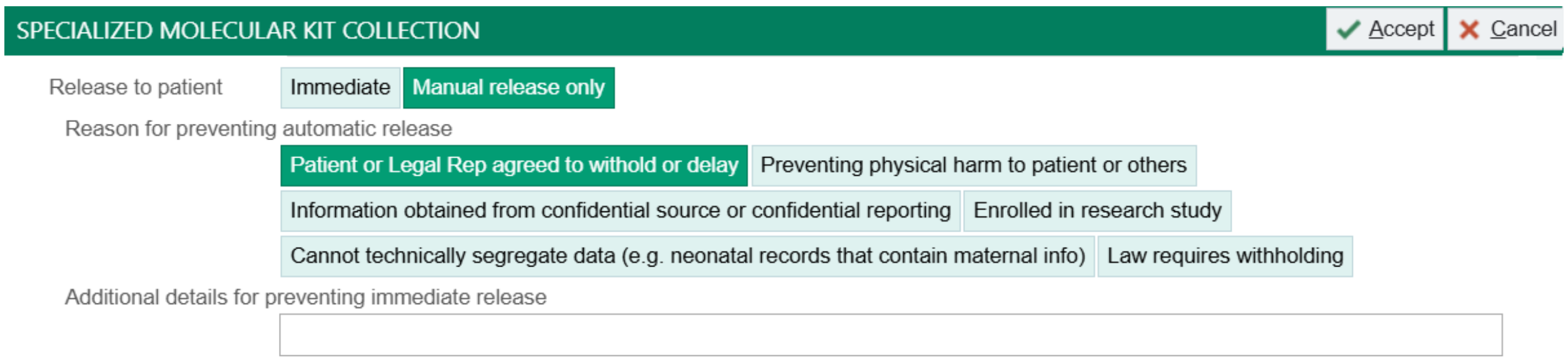

B. Screenshot of test results that were initially selected as "manual release only" and have not been released yet.

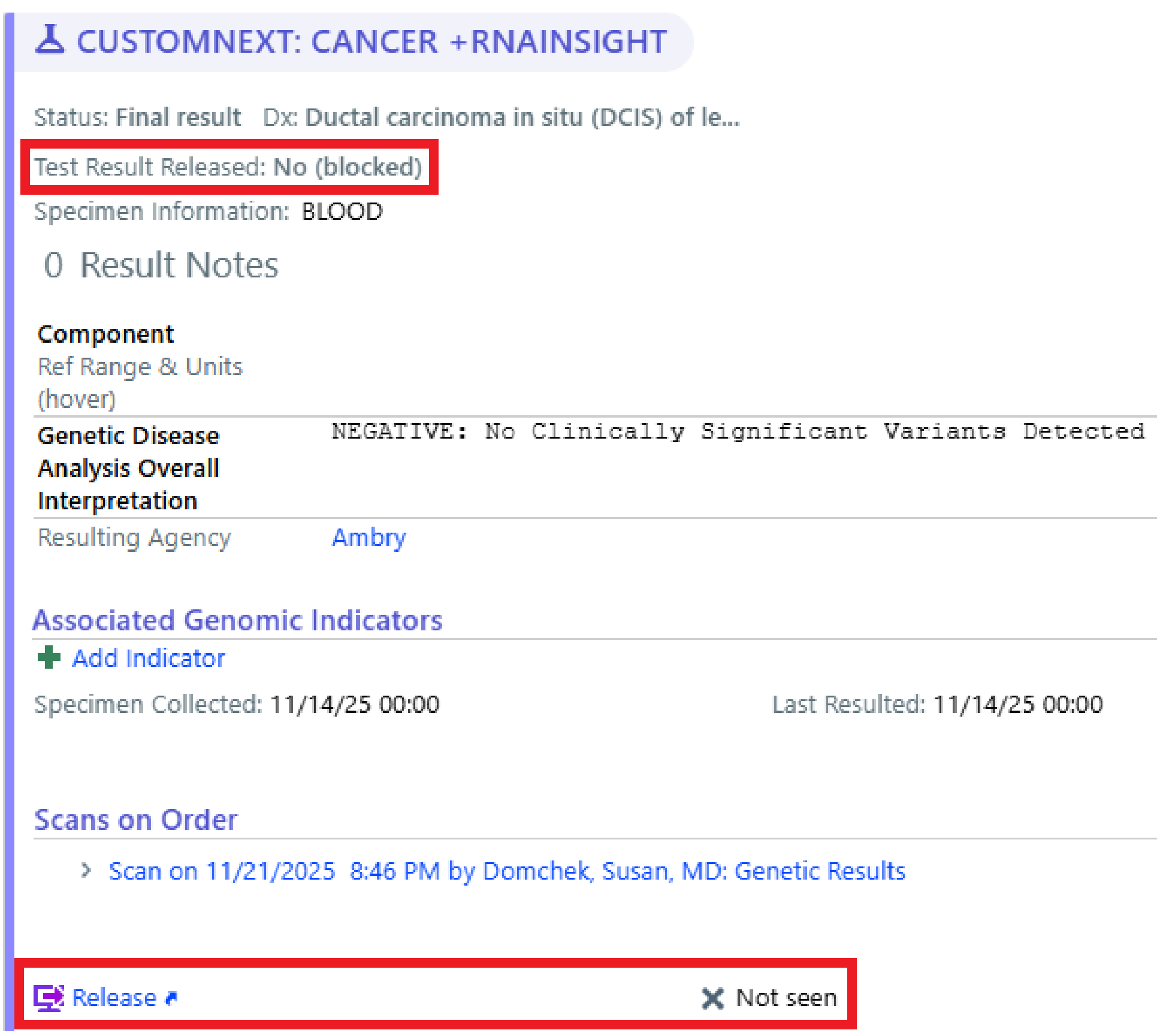

C. Screenshot of test results that were initially selected as “immediate release” and have already been viewed by the patient.

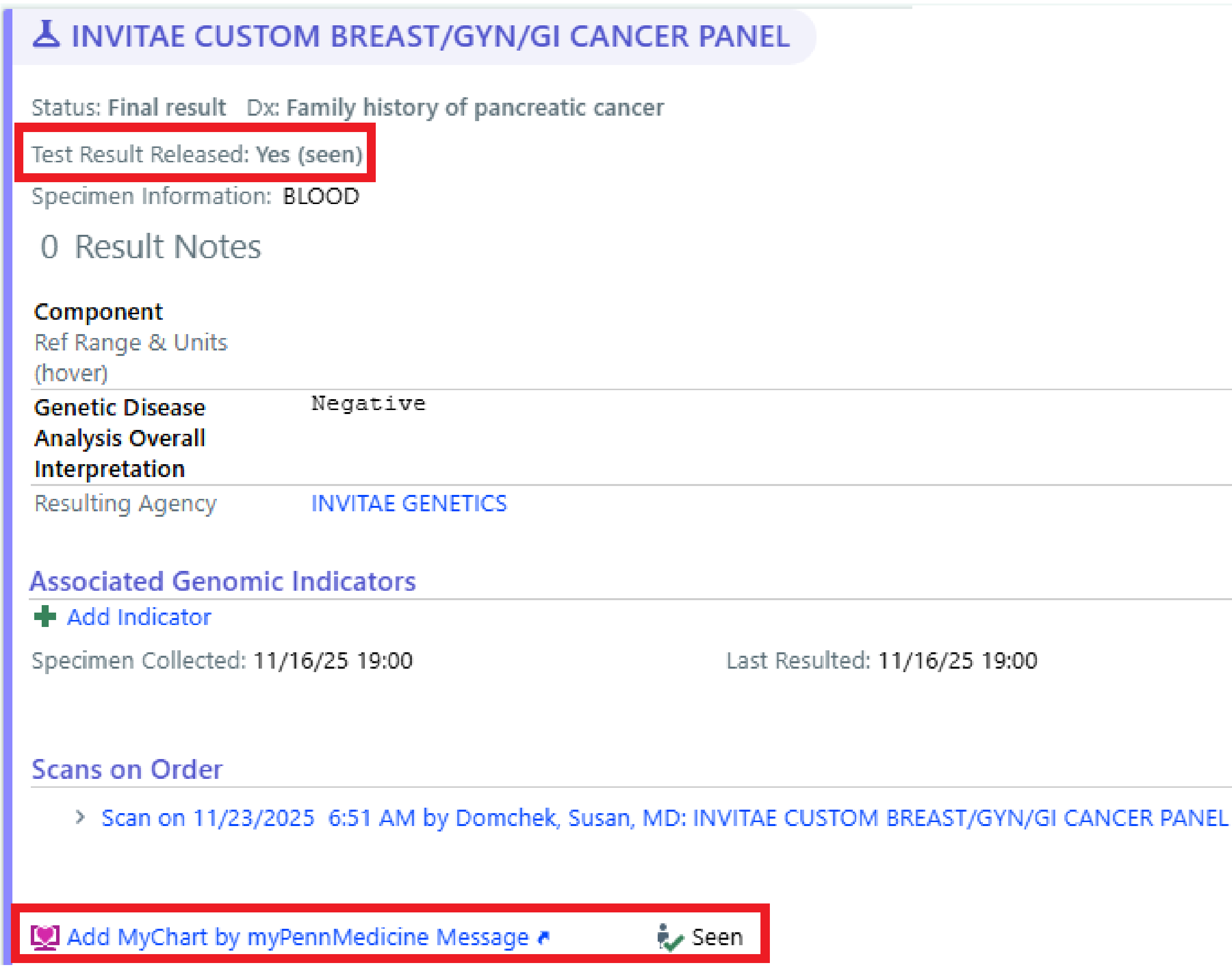

INVITAE CUSTOM BREAST/GYN/GI CANCER PANEL

Status: **Final result** Dx: **Family history of pancreatic cancer**

Test Result Released: **Yes (seen)**

Specimen Information: BLOOD

0 Result Notes

Component<br>Ref Range & Units<br>(hover)	
**Genetic Disease Analysis Overall Interpretation**	Negative
Resulting Agency	INVITAE GENETICS

Associated Genomic Indicators

Add Indicator

Specimen Collected: 11/16/25 19:00 Last Resulted: 11/16/25 19:00

Scans on Order

Scan on 11/23/2025 6:51 AM by Domchek, Susan, MD: INVITAE CUSTOM BREAST/GYN/GI CANCER PANEL

Add MyChart by myPennMedicine Message Seen

Online Resource 2: Participant survey

Note: Color coding represents skip logic.

> Yellow: For people who select hereditary cancer for type of testing
> Teal: For people who select VUS for result type
> Green: For those who saw results prior to context provided by HCP

The questions below relate to the genetic testing you had recently to see if you have, or are a carrier for, a hereditary condition. This is also called "germline" genetic testing and is done on a blood or saliva sample. These questions do not apply to tumor (somatic) testing of cancer.

1. What was the main reason you had the recent genetic test?
   - Personal history of a condition with no known genetic cause
   - Family history of a condition with no known genetic cause
   - A family member tested positive for a gene mutation
   - Other (please specify)

2. What type of health condition was the genetic testing related to?
   - Hereditary cancer
   - Neurologic condition
   - Heart condition
   - Immune condition
   - Kidney condition
   - Prenatal screening
   - Other (please specify)
   - I don't know

3. What was the result of your genetic test? (select all that apply)
   - Negative (meaning the test results did not show any genetic changes)
   - Positive (meaning a pathogenic or likely pathogenic gene mutation was identified)
     - Please specify the gene if you know it:
   - Variant of uncertain significance
   - Other (please specify) _______________
   - I don't know

4. There is currently no plan to change my medical care because of this result.
   - True
   - False
   - I don't know

5. I have been told that my family members should have genetic testing because of my test result.
   - True
   - False
   - I don't know

6. My test result means a hereditary cause for my personal or family history of cancer was identified.
    - o True
    - o False
    - o I don't know

7. There are other genes related to cancer risk that were not included on my test.
    - o True
    - o False
    - o I don't know

8. It is common to find a variant of uncertain significance.
    - o True
    - o False
    - o I don't know

9. The meaning of my variant may change in the future as more information is learned.
    - o True
    - o False
    - o I don't know

10. Most uncertain variants end up being harmful (disease causing) once more information is learned.
    - o True
    - o False
    - I don't know
11. Did you meet with a genetic counselor **before** your genetic test was ordered?
    - o Yes
    - o No
    - o I don't know

12. Did you receive enough information about genetic testing before moving forward with testing?
    - o No, I didn't know that genetic testing was being performed
    - o No, I didn't understand what doing genetic testing meant
    - o Yes, but more info would have helped
    - o Yes
    - o Not sure

13. How did you **first** learn about your recent genetic test results?
    - o Telephone call with a healthcare provider
    - o Video/telehealth call with a healthcare provider
    - o MyChart message from a healthcare provider
    - o Saw my result in MyChart before any communication from a healthcare provider
    - o Through a third-party lab portal before any communication from a healthcare provider (Examples: Invitae, Myriad, Natera, etc.)

- Other (please specify)

14. Was this how you expected to learn about your genetic test results?
    - Yes
    - No
    - Other (please specify)

15. If a healthcare provider reviewed your results with you, please indicate how (select all that apply):
    - MyChart message
    - Phone call
    - At an in-person visit
    - At a telehealth (virtual) visit
    - Does not apply; a health care provider did not review these results with me
    - Other (please specify)

16. At any point, did you discuss your results with a genetic counselor?
    - Yes
    - No
    - I don't know
    - Other (please specify)

17. Where did you go for more information about the result of your genetic test? Select all that apply:
    - ☐ Family member/relative
    - ☐ Friend
    - ☐ A health care provider, such as a genetic counselor, doctor or nurse
    - ☐ Someone I work with
    - ☐ Social media (Facebook, Twitter, Instagram, etc.)
    - ☐ An internet search (please specify)
    - ☐ Other (please specify)
    - ☐ I did not seek additional information

18. Did looking at the genetic test results before your provider had a chance to review it with you make you more or less worried about your health?
    - Much less worried
    - Less worried
    - No change
    - More worried
    - Much more worried
    - I was never worried

19. If you had a reason to pursue additional genetic testing in the future, how would you like to **first** learn about your genetic test results?
    - ☐ Phone call from your provider
    - ☐ Appointment with your provider
    - ☐ View it on MyChart, with a message from your provider

- ◻ View it on MyChart, without any provider review or explanation
- ◻ Other (please specify)
- ◻ Don't know/not sure

When you **FIRST** learned your genetic test result:

20. How anxious or nervous did you feel about your genetic test result?
    - ○ Not at all
    - ○ A little
    - ○ Somewhat
    - ○ A good deal
    - ○ A great deal

21. How relieved did you feel about your genetic test result?
    - ○ Not at all
    - ○ A little
    - ○ Somewhat
    - ○ A good deal
    - ○ A great deal

22. How uncertain did you feel about what your genetic test result means for you?
    - ○ Not at all
    - ○ A little
    - ○ Somewhat
    - ○ A good deal
    - ○ A great deal

23. How uncertain did you feel about what your genetic test result means for your child(ren) and/or family's risk of disease?
    - ○ Not at all
    - ○ A little
    - ○ Somewhat
    - ○ A good deal
    - ○ A great deal

24. How much did you feel that you understood clearly your choices for care based on the genetic test result?
    - ○ Not at all
    - ○ A little
    - ○ Somewhat
    - ○ A good deal
    - ○ A great deal

25. Is there anything else you'd like to share regarding your experience with receiving your genetic test results?

26. Please enter your current age: ____________

27. Which option best describes your gender?
    - o Male
    - o Female
    - o Non-binary
    - o Other

28. What do you consider to be your racial background? Check all that apply.
    - □ American Indian, Alaskan Native or Indigenous Peoples of Canada
    - □ Asian
    - □ Black, African American or of African descent
    - □ Jewish
    - □ Hispanic or Latine
    - □ Middle Eastern or North African (MENA)
    - □ Native Hawaiian or Other Pacific Islander
    - □ White
    - □ Prefer to self-describe
    - □ Prefer not to answer

29. What is the highest grade or level of school that you have completed?
    - o 8th grade or less
    - o Some high school, but did not graduate
    - o High school graduate or GED
    - o Some college, technical school, or 2-year degree
    - o 4- year college graduate
    - o Some graduate school
    - o Masters or Doctoral degree

30. Have you worked in a health care organization?
    - o Yes, in a clinical role
    - o Yes, in a non-clinical role
    - o No

31. What is your total yearly estimated household income?
    - o Less than $50,000
    - o 50,000-$150,000
    - o More than $150,000
    - o Prefer not to answer
